# Could She Be Autistic? Exploring Gender Differences in Camouflaging and Pragmatics in Autism and Borderline Personality Disorder

**DOI:** 10.1002/cpp.70210

**Published:** 2026-01-07

**Authors:** Raquel Sotos Gracia, Patricia López Resa, Andreea Ioana María Escudero Timerman, Seila María García Gómez

**Affiliations:** ^1^ Department of Psychology Faculty of Health Sciences University of Castilla‐La Mancha Toledo Spain

**Keywords:** autism, borderline personality disorder, differential diagnosis, gender, pragmatics, social camouflaging

## Abstract

This study explores the relationship between social camouflaging and pragmatic competence in adults diagnosed with autism spectrum disorder (ASD) and borderline personality disorder (BPD), with a particular focus on gender. It is based on the hypothesis that camouflaging contributes to under or misdiagnosis, especially in women and gender‐diverse individuals. A total of 225 adults participated in a cross‐sectional online survey, completing the Camouflaging Autistic Traits Questionnaire (CAT‐Q) and the Pragmatic Awareness Questionnaire (PAQ). Participants were grouped based on clinical diagnosis (ASD or BPD) and self‐identified gender (women, men and gender‐diverse). Among women, no significant differences in camouflaging scores were found between the ASD and BPD groups, suggesting the use of similar adaptation strategies that may obscure clinical differentiation. In contrast, among men, camouflaging and pragmatic deficits were more distinctly associated with autistic traits. No substantial differences were observed among gender‐diverse participants, highlighting the influence of contextual and identity‐related factors. Findings emphasize the importance of integrating detailed pragmatic assessments and adopting gender‐sensitive approaches in the differential diagnosis of ASD and BPD. Such strategies may help reduce misdiagnosis and improve recognition of autistic traits, particularly in populations that tend to camouflage more effectively.

## Summary

- Social camouflaging can obscure the distinction between autism and borderline personality disorder, particularly among women, potentially contributing to underdiagnosis or misdiagnosis.
- Pragmatic language differences appear more pronounced in men, suggesting greater diagnostic value of pragmatic assessment in male populations.
- In gender‐diverse individuals, camouflaging may reflect sociocultural pressures and identity‐related factors rather than diagnostic profiles alone, underscoring the need for gender‐sensitive assessment approaches.
- Sole reliance on self‐report measures may miss underlying communication challenges; combining developmental history, observational data and multiple informants can enhance diagnostic accuracy.

## 1 Introduction

Autism spectrum disorder (ASD) is a neurodevelopmental condition involving differences in social communication and restricted or repetitive patterns of behaviour (DSM‐5‐TR, 2022). Historically, ASD diagnosis has been based on what is known as the classic phenotype (Lai et al. 2015), which was primarily developed through research with male children. This male‐biassed model has contributed to significant gender disparities in diagnosis, with estimates suggesting that only one woman is diagnosed for every four men (Loomes et al. 2017). Already by the early 2000s, researchers such as Piven (2001) began to describe a broader autism phenotype characterized by subtler and more heterogeneous traits, particularly in females. Consequently, many autistic women remain undiagnosed or are misdiagnosed with conditions such as borderline personality disorder (BPD) (McQuaid et al. 2024). Recent research also highlights that diagnostic complexity may increase when ASD co‐occurs with other mental health conditions, particularly personality and affective disorders, with reported comorbidity rates ranging between 15% and 30% (Dell'Osso et al. 2023; Rydén and Bejerot 2008).

BPD, in contrast, is defined as a personality disorder marked by emotional instability, difficulties with affect regulation, identity disturbances and conflicted interpersonal relationships (DSM‐5‐TR, 2022). Unlike ASD, BPD is more frequently diagnosed in women, with an approximate ratio of three women for every man. Moreover, several studies suggest a significant comorbidity between ASD and BPD (Dell'Osso et al. 2023). Petersen et al. (2016) highlight a notable symptom overlap between the two disorders, particularly in areas such as mentalization, emotional state interpretation and emotional regulation—posing a substantial clinical challenge. The similarities between ASD and BPD are especially evident in difficulties related to social interaction and self‐perception (Petrolini and Schmidt‐Boddy 2025). However, while both conditions may present comparable challenges, other studies suggest that their origins and clinical manifestations diverge (Dudas et al. 2017; McQuaid et al. 2024). In ASD, theory of mind impairments appears to stem from atypical social processing during developmental stages, whereas in BPD, such impairments are associated with early traumatic experiences and unstable mentalization, marked by fluctuating and often distorted interpretations of others' intentions (Dell'Osso et al. 2023).

Given these shared and distinct underlying mechanisms, various studies have explored the relationship between the two disorders. Due to the aforementioned overlap, Dudas et al. (2017) identified a high prevalence of autistic traits in women diagnosed with BPD, suggesting that some cases might correspond to undiagnosed ASD. In their study, they compared three clinical groups: individuals with ASD, individuals with BPD and a comorbid group with both diagnoses. Using instruments such as the Autism Spectrum Quotient (AQ; Baron‐Cohen et al. 2001), the Empathy Quotient (EQ; Baron‐Cohen and Wheelwright 2004) and the Systemizing Quotient‐Revised (SQ‐R; Baron‐Cohen et al. 2003), they observed significantly elevated scores for autistic traits and systemizing in both the BPD and comorbid groups. However, the BPD group did not exhibit the same empathy deficits observed in the ASD group, suggesting a distinct yet convergent symptom profile. Other researchers hypothesize that some women with ASD, when exposed to adverse environmental factors or prolonged experiences of social invalidation, may develop personality patterns that meet diagnostic criteria for BPD. In such cases, the symptom overlap may not reflect true comorbidity but rather a complex differential diagnosis, where autistic features have previously gone unrecognized or have been masked through compensatory strategies (Dell'Osso et al. 2023; McQuaid et al. 2024). This underscores the importance of thorough clinical assessment that considers developmental history and the impact of camouflaging, particularly in women with ASD.

From a theoretical perspective, camouflaging has been proposed as a central construct to better understand the diagnostic challenges in differentiating ASD from BPD, especially in females. In the case of ASD, the *camouflaging hypothesis* (Hull et al. 2017) suggests that many autistic women develop conscious, structured strategies to hide their social difficulties—such as mimicking neurotypical behaviours, explicitly learning social norms and employing rehearsed conversational scripts (Hull et al. 2020; Alaghband‐Rad et al. 2023). Studies such as Dean et al. (2017) further indicate that these strategies are more frequent and sophisticated in women, contributing to underdiagnosis. These compensatory mechanisms are typically motivated by the desire to achieve social integration and avoid rejection. However, research also warns of the significant emotional cost associated with sustained camouflaging efforts. At the same time, recent work presents a more nuanced view of camouflaging: It can confer short‐term advantages (e.g., facilitating social inclusion, reducing immediate stigma exposure or enabling alternative ways of engaging) while still carrying longer term costs. A recent systematic review reported such short‐term benefits alongside impacts on identity, delayed or missed diagnosis and mental‐health strain (Summerill and Summers 2025). Qualitative and conceptual work has likewise framed camouflaging as a “two‐edged” adaptation shaped by anxiety and public stigma rather than mere role‐playing, helping some autistic adults to participate socially even as it increases exhaustion and burnout risks (Pyszkowska 2025). In line with the reviewer's suggestion, we therefore adopt a balanced stance: Camouflaging may be both adaptively useful in context and a source of cumulative psychological burden.

In contrast, emotional regulation patterns in BPD may result in behaviours resembling social camouflaging, though these are often reactive and unconscious, rather than deliberate or strategic (Camañero Ballestar et al. 2023). Although the term *camouflaging* has not been explicitly applied to BPD in the literature, Petrolini and Schmidt‐Boddy (2025) propose that the phenomenon described as facade (façade) could be interpreted as a distinct manifestation of camouflaging in individuals with BPD. This concept refers to a self‐presentation strategy in which the individual attempts to project a coherent, competent or socially acceptable image despite having a fragmented or diffuse internal identity (Jørgensen and Bøye 2022). In this sense, such individuals may engage in social camouflaging behaviours similar to those observed in autistic women. Furthermore, some studies suggest that misdiagnosis of BPD may inadvertently reinforce masking behaviours through unsuitable or misdirected intervention approaches (Cassidy et al., 2018).

These differences in camouflaging profiles not only affect social and emotional perception but may also influence language use, representing another potential axis for differentiating the two conditions (Muriel et al. 2023; Ying Sng et al. 2018). In ASD, masking may obscure pragmatic language difficulties, complicating the identification of deficits in social language use (Hull et al. 2017). These difficulties include turn‐taking, discourse coherence and the interpretation of communicative intent, all of which impact social reciprocity and fluency (Muñoz Sánchez et al. 2024). Regarding BPD, some studies report that communicative alterations stem from emotional instability and shifting perceptions of others (Weiner et al. 2023), while others have documented self‐perceived pragmatic difficulties—such as discourse coherence, adaptation to interlocutors and use of conversational inferences—that are consistent with external evaluations (Muriel et al. 2023). Additionally, Montigny‐Malenfant et al. (2013) found that women with BPD tend to exhibit communication styles marked by criticism, emotional reactivity and disruption of conversational flow, particularly in significant interpersonal contexts such as romantic relationships. In both cases, these patterns may contribute to clinical perceptions of disorganized communication and highlight how camouflaging strategies alter the presentation of communicative difficulties—posing an additional challenge in differential diagnosis and reinforcing the need for detailed linguistic‐pragmatic assessment for each condition.

In this context, understanding how communicative profiles and camouflaging strategies affect the differential diagnosis between ASD and BPD is crucial. However, most studies to date have focused on general clinical descriptions rather than on systematic comparisons using specific assessment tools (Dell'Osso et al. 2023; Dudas et al. 2017; McQuaid et al. 2024).

To address these gaps, the current study aimed to examine the role of gender in shaping social camouflaging and pragmatic communication in individuals diagnosed with ASD and BPD. Specifically, it aimed the following:

1. Compare levels of social camouflaging across ASD and BPD groups, considering gender differences
2. Assess pragmatic communication profiles in both conditions to identify patterns of overlap and divergence.
3. Explore the interaction between camouflaging and pragmatic competence, particularly in women and gender‐diverse individuals.
4. Provide empirical data to support more accurate, gender‐informed differential diagnosis between ASD and BPD.

## 2 Method

### 2.1 Participants

A total of 368 adults initially responded to the study invitation. Of these, only participants who provided written documentation of a formal clinical diagnosis issued by a licensed mental‐health professional were included in the final sample. Individuals who reported only self‐diagnosis were excluded.

After verification of diagnostic documentation, the final sample comprised 225 adults with a confirmed diagnosis of either ASD or BPD, ranging in age from 18 to 43 years. All participants presented written documentation of a formal diagnosis issued by a licensed mental‐health professional. Individuals who reported only a self‐diagnosis were excluded from the study.

Participants were recruited from across Spain through online dissemination via social media platforms and clinical networks. The sample was geographically diverse, with the largest proportions from Andalucía (17.3%), Cataluña (16.4%), Madrid (14.2%) and the Comunidad Valenciana (10.7%), followed by smaller representations from Galicia, Canarias, Castilla y León, Castilla‐La Mancha and the País Vasco, among others. This distribution broadly reflects the population density and digital accessibility of these regions.

The BPD group included 103 participants (52 women, 39 men and 12 gender‐diverse individuals) with a mean age of 29.9 years and a standard deviation (SD) of 7.45.

The ASD group was composed of 122 participants (63 women, 47 men and 12 individuals with diverse gender identities), with a mean age of 29.7 years and an SD of 7.75. Gender‐diverse participants included individuals who self‐identified in writing as nonbinary or gender‐fluid.

### 2.2 Instruments

Two self‐report instruments were used to assess the central variables of the study: the use of social camouflaging strategies and pragmatic awareness in communicative contexts. Both instruments were developed for adult populations and have been previously employed in studies with similar objectives.

The first instrument was the Camouflaging Autistic Traits Questionnaire (CAT‐Q), developed by Hull et al. (2019). This instrument assesses the frequency with which individuals use strategies to conceal or compensate for traits associated with ASD in social environments. It consists of 25 items grouped into three subscales: Compensation (e.g., mimicking social scripts), Masking (suppressing behaviours perceived as atypical) and Assimilation (efforts to fit in socially). Each item is rated on a 7‐point Likert scale ranging from ‘strongly disagree’ to ‘strongly agree’. Scores can be interpreted through the individual subscales or via a global total score. Four items (3, 10, 11 and 15) require reverse scoring to control for response bias. Although no official Spanish adaptation exists, the CAT‐Q has been translated and applied in prior research with Spanish‐speaking populations, such as the preliminary validation conducted by Mosquera et al. (2022). In its original version, the questionnaire shows excellent internal consistency (*α* = 0.94 for the total scale; between 0.85 and 0.92 for the subscales), and it takes approximately 10–15 min to complete.

The second instrument was the Pragmatic Awareness Questionnaire (Cuestionario de Conciencia Pragmática; CCP) developed by Rodríguez Muñoz (2012). This self‐administered questionnaire, originally created in Spanish, evaluates individuals' critical and metapragmatic awareness regarding language use in various communicative contexts. It encompasses three primary dimensions of pragmatic competence: the communicative dimension, which evaluates discourse production through aspects such as body posture, gesture use, intonation and vocal volume; the textual dimension, assessing coherence, cohesion and message organization; and the interactive dimension, which examines turn‐taking, conversation maintenance and interpretation of nonverbal cues during social interaction. The CCP comprises 26 statements, each rated on a 5‐point Likert scale (from ‘very poorly’ to ‘very well’). The scale demonstrates excellent internal reliability (*α* = 0.972), although it lacks normative benchmarks. Estimated completion time is 10–15 min.

### 2.3 Procedure

Participants were recruited through outreach to mental health organizations and via the dissemination of the study through social media, using a nonprobabilistic convenience sampling.

Individuals expressing interest in participating were invited to complete a digital informed consent form, which also included a brief sociodemographic section collecting data such as age, gender identity, place of residence, clinical diagnosis and the credentialed professional who issued the diagnosis.

Following consent, participants completed a structured interview lasting approximately 25 min. The purpose of this interview was to verify that all inclusion and exclusion criteria were met. This included confirmation of a formal clinical diagnosis of ASD or BPD issued by a qualified professional and functional proficiency in Spanish necessary to understand the study materials.

During this interview, participants were asked to present written evidence of their diagnosis (e.g., a clinical report or certificate) issued by a licensed mental health professional. All interviews were conducted by a trained member of the research team following a standardized protocol to ensure consistency across cases. Participants who reported a self‐diagnosis of autism were excluded from the present study. The interview also served to clarify questions, ensure adequate comprehension of the study's aims and promote participant engagement.

Upon verification of eligibility, participants were provided with a link to complete the questionnaires online via the Microsoft Forms platform. The form included both standardized instruments: the CAT‐Q and the CCP, which participants were instructed to complete independently immediately following the interview session.

To ensure the validity and reliability of the collected data, several control measures were implemented. Incomplete responses and those with invalid patterns—such as uniform answers across all items—were excluded. Additionally, response timestamps were reviewed to identify atypical completion behaviours.

All data were anonymized, coded and securely stored in compliance with current data protection regulations. Participation was entirely voluntary, with no financial compensation offered, and confidentiality was assured at all times.

### 2.4 Data Analysis

Data analysis was conducted using Python. Initially, descriptive statistics were calculated for all variables of interest. For quantitative variables, mean, median, standard deviation and interquartile range were reported. For categorical variables, absolute and relative frequencies were obtained.

Next, the normality of the quantitative variables was assessed through multiple procedures. Shapiro–Wilk tests, skewness and kurtosis coefficients and graphical methods (e.g., histograms, Q–Q plots) were used to determine distribution patterns. A variable was considered to approximate a normal distribution if skewness and kurtosis fell within ±2.

Results indicated that the variables did not follow a normal distribution (*p* < 0.05 or skewness/kurtosis outside expected ranges). Consequently, nonparametric methods were applied for inferential analysis.

To compare independent groups, the Mann–Whitney *U* test was used. No corrections for multiple comparisons were applied, given the exploratory nature of the study. A significance level of *p* < 0.05 was adopted for all tests.

## 3 Results

With the aim of exploring potential differences in social camouflaging and pragmatic competence, both within and between clinical groups, various statistical analyses were conducted with diagnosis and gender considered as the main variables.

First, differences within each clinical group (ASD and BPD) were analysed based on gender. Overall, statistically significant differences (*p* < 0.05) were found in most of the variables assessed. However, in the ASD group, no significant differences (*p* > 0.05) were observed in items related to nonverbal language, discourse cohesion and turn‐taking management. Similarly, in the BPD group, no significant differences (*p* > 0.05) were found in variables related to facial expression, eye gaze, communicative context adequacy and thematic progression.

These differences (and the absence of differences) justified specific intergroup comparisons by gender, organized into three pairs: women with ASD vs. women with BPD, men with ASD vs. men with BPD and individuals with diverse gender identities diagnosed with ASD vs. those diagnosed with BPD.

### 3.1 Comparison Between Women Diagnosed With ASD and BPD

Regarding the CAT‐Q (Hull et al. 2019), the statistical analysis did not reveal significant differences in the total score or in any of its subscales: masking, compensation and assimilation (*p* > 0.05).

In contrast, results obtained through the CCP (Rodríguez Muñoz 2012) showed statistically significant differences (*p* < 0.05) in two specific dimensions: syntactic cohesion (*p* < 0.05) and the maxim of relevance (*p* < 0.05). For the remaining pragmatic variables assessed (intelligibility and paralinguistic features, use of interpersonal space, leg and arm kinesics, body posture, gesturing, facial expression, eye gaze, lexical and morphological cohesion, lexical competence, interpretation of ambiguous expressions, understanding of irony and humour, textual coherence, communicative adequacy, thematic progression, thematic change and maintenance, turn‐taking management and quantity), no significant differences were observed between the two groups (*p* > 0.05).

A post hoc power analysis (jamovi, two‐tailed *t*‐test, *α* = 0.05, *d* = 0.50) indicated a power of 0.76 for this comparison, suggesting adequate sensitivity to detect medium effects.

### 3.2 Comparison Between Men Diagnosed With ASD and BPD

Regarding the CAT‐Q (Hull et al. 2019), no significant differences (*p* > 0.05) were found between the groups on any of the subscales: compensation, masking and assimilation. However, the total questionnaire score showed a statistically significant difference between the two groups (*p* < 0.05).

As for self‐perceived pragmatic competence indicators assessed through the CCP (Rodríguez Muñoz 2012), statistically significant differences (*p* < 0.05) were found in the following dimensions: facial expression (*p* < 0.05), lexical cohesion (*p* < 0.05), understanding of irony (*p* < 0.05), understanding of humour (*p* < 0.05), morphological cohesion (*p* < 0.05), linguistic appropriateness to the situation or interlocutor (*p* < 0.05) and the maxim of quantity (*p* < 0.05). In the rest of the pragmatic variables assessed (intelligibility and paralinguistic features, use of interpersonal space, leg and arm kinesics, gesturing, eye gaze, lexical competence, interpretation of ambiguous expressions, syntactic cohesion, textual coherence, thematic progression, thematic change and maintenance, turn‐taking management and relevance), no significant differences were observed between the groups (*p* > 0.05).

The post hoc power analysis (jamovi, two‐tailed *t*‐test, *α* = 0.05, *d* = 0.50) yielded a power of 0.63 for this comparison, reflecting moderate sensitivity to detecting medium‐sized effects.

### 3.3 Comparison Between Gender‐Diverse Individuals Diagnosed With ASD and BPD

In the comparison between individuals with diverse gender identities diagnosed with ASD and those diagnosed with BPD, no statistically significant differences were observed in the total CAT‐Q score (Hull et al. 2019) or in any of its subscales (compensation, masking and assimilation) (*p* > 0.05).

Regarding aspects related to pragmatic competence assessed with the CCP (Rodríguez Muñoz 2012), only one significant difference was observed in the item corresponding to textual superstructure coherence (*p* < 0.05). The remaining dimensions evaluated (intelligibility and paralinguistic features, use of interpersonal space, leg and arm kinesics, body posture, gesturing, facial expression, eye gaze, lexical and morphosyntactic cohesion, lexical competence, interpretation of ambiguous expressions, understanding of irony and humour, linguistic appropriateness, thematic progression, thematic change and maintenance, turn‐taking management, relevance and quantity) did not show statistically significant differences between the two groups (*p* > 0.05).

The achieved statistical power for this subgroup (ASD = 12, BPD = 12; *α* = 0.05, *d* = 0.50) was 0.22, indicating low sensitivity to effects of medium magnitude due to the small sample size.

The results of the post hoc comparisons between the ASD and BPD diagnostic groups, stratified by gender identity, are presented in Table 1.

**TABLE 1:** Comparison results between ASD and BPD diagnostic groups, stratified by gender identity.

	Women		Men		Gender‐diverse individuals	
Pragmatic awareness questionnaire	*h*	*p*	*h*	*p*	*h*	*p*
Intelligibility	1634	0.981	740	0.409	57.5	0.380
Paralanguage	1440	0.240	807	0.862	69.5	0.905
Interactional proxemics	1498	0.405	729	0.329	56.0	0.343
Social proxemics	1465	0.300	797	0.790	61.0	0.520
Body posture	1602	0.832	678	0.153	72.0	0.163
Movements of feet and legs	1437	0.227	790	0.730	63.0	0.620
Movements of hands and arms	1487	0.369	785	0.695	57.5	0.380
Gestures	1445	0.250	755	0.487	69.5	0.905
Facial expression	1429	0.215	599	0.029*	58.0	0.405
Gaze	1390	0.138	794	0.754	42.5	0.075
Lexical cohesion	1443	0.246	321	< 0.001***	53.5	0.287
Lexical competence	1448	0.256	334	< 0.001***	68.0	0.830
Interpretation of ambiguous expressions	1609	0.863	802	0.823	60.5	0.500
Comprehension of irony	1592	0.786	207	< 0.001***	67.0	0.782
Comprehension of humour	1402	0.161	123	< 0.001***	62.5	0.549
Morphological cohesion	1460	0.288	415	< 0.001***	58.5	0.441
Syntactic cohesion	1284	0.042*	738	0.398	70.0	0.927
Superstructural coherence	1510	0.444	637	0.066	14.0	< 0.001***
Linguistic appropriateness	1455	0.272	261	< 0.001***	67.5	0.806
Macrostructural coherence	1461	0.295	775	0.615	64.0	0.644
Thematic drift	1386	0.133	737	0.371	68.0	0.833
Thematic progression	1557	0.632	770	0.591	64.5	0.667
Turn‐taking agility	1539	0.557	706	0.242	69.5	0.902
Maxim of relevance	1268	0.032*	766	0.560	65.5	0.721
Maxim of quantity	1512	0.465	133	< 0.001***	64.0	0.646
CAT‐Q						
Compensation	1513	0.479	768	0.598	61.5	0.561
Masking	1475	0.357	754	0.511	42.5	0.091
Assimilation	1561	0.664	749	0.483	63.0	0.620
Total score	1559	0.653	606	0.043*	45.5	0.132

* *p* < 0.05.

** *p* < 0.01.

*** *p* < 0.001.

## 4 Discussion

The results revealed a mixed pattern in which some dimensions of the CAT‐Q (Hull et al. 2019) and the Pragmatic Awareness Questionnaire (CCP; Rodríguez Muñoz 2012) differed significantly across genders within each clinical group, while others showed no substantial variation. Overall, these results should be interpreted with caution, as the large number of subscales analysed increases the likelihood of finding isolated differences by chance. Rather than pointing to a consistent gender effect, these findings suggest that the expression of camouflaging and pragmatic difficulties may depend on how social and diagnostic expectations interact with gendered adaptation strategies. Accordingly, interpretations remain tentative and warrant replication in large, independent samples. This has direct implications for clinical practice, as it challenges the assumption that gender‐neutral criteria can capture the diversity of communicative profiles in neurodivergent populations.

In the case of ASD, prior studies have indicated that men often display a more restricted camouflaging profile, primarily oriented toward behavioural control (Cage and Troxell‐Whitman 2019), whereas women tend to develop more complex and socially elaborated strategies (Hull et al. 2019). Regarding BPD, the literature has described variation in emotional expression and conversational patterns as a function of gender, particularly in relation to identity construction and affect regulation (Camañero Ballestar et al. 2023; Jørgensen and Bøye 2022). However, the lack of significant differences in variables such as facial expression or contextual appropriateness among participants with BPD suggests that communicative irregularities in this group may stem more from emotional instability than from underlying neurodevelopmental differences—posing a challenge for differential diagnosis when such signs are interpreted without context. Future studies should therefore explore more directly how gender moderates pragmatic functioning and camouflaging in both conditions.

Stratified gender‐based comparisons revealed distinct camouflaging behaviours and communicative self‐perceptions. When comparing women with ASD and women with BPD, no significant differences were found in any of the camouflaging dimensions measured. This lack of differentiation may suggest a substantial overlap in how camouflaging manifests among women across diagnostic categories, possibly reflecting shared socialization processes and adaptive pressures. From a diagnostic standpoint, this overlap may result in autistic women being overlooked or misclassified, particularly when their adaptation strategies succeed in simulating neurotypical interaction patterns. Several studies have emphasized that the symptoms of autistic women may go unnoticed due to their use of more sophisticated social adaptation strategies, complicating accurate identification (Hull et al. 2019; Lai et al. 2015). In this light, apparent social competence should not be assumed to exclude ASD, especially when not supported by consistent developmental history or stable interpersonal functioning.

Although the literature on camouflaging in BPD is still limited, recent studies have begun to explore phenomena consistent with this concept. Petrolini and Schmidt‐Boddy (2025) proposed the notion of façade, defined as a self‐presentation strategy motivated by a need for control in the face of internal emotional instability—functioning similarly to camouflaging in individuals with ASD. This phenomenological overlap may account for the similarities in CAT‐Q scores and the lack of diagnostic differentiation between both clinical groups. If both conditions involve adaptive behaviours that obscure core features, distinguishing between them requires moving beyond surface‐level presentation and emphasizing developmental markers, interpersonal history and the consistency of traits over time. Future research should explore camouflaging in BPD more deeply, examining its mechanisms, trajectories and clinical implications. Developing assessment tools specifically designed to capture camouflaging behaviours in this population could help improve differential diagnosis and prevent clinical misinterpretation.

A similar pattern was observed in communicative self‐perception, where significant differences emerged only in the dimension related to the maxim of relevance. This limited differentiation could reflect the effect of camouflaging (Dean et al. 2017; Hull et al. 2017), which may influence individuals' subjective assessments of their own pragmatic abilities (Livingston et al. 2019). Thus, women with BPD may rate their pragmatic performance similarly to women with ASD—not because their communicative profiles are equivalent but because adaptation mechanisms distort self‐evaluation, masking real differences. This convergence complicates diagnostic procedures that rely on introspective self‐report alone and underlines the importance of integrating developmental interviews and third‐party observations.

In contrast, when comparing men with BPD and those with ASD, statistically significant differences in camouflaging were found, with higher scores in the ASD group. This suggests that in male populations, social camouflaging strategies are more strongly associated with ASD traits. However, these gender‐specific findings should be regarded as preliminary require further investigation to determine their robustness and clinical meaning. This aligns with previous literature describing that men with ASD often show a less elaborate or more restricted camouflaging profile compared to women (Cage and Troxell‐Whitman 2019; Hull et al. 2019) and tend to compensate for social challenges mainly through behavioural control. Accordingly, the difference observed in the CAT‐Q total score may reflect a lower prevalence of camouflaging in men with BPD or, alternatively, a more overt expression of autistic traits in men, which facilitates clinical identification. This contrast highlights the gendered visibility of autistic traits and the varying diagnostic thresholds across genders.

Unlike what was observed in women, results for men showed marked discrepancies in pragmatic abilities, particularly in dimensions associated with core ASD symptoms (Reindal et al. 2023), such as facial expression, comprehension of irony and humour, lexical and morphological cohesion, contextual appropriateness and verbal quantity. These findings, though exploratory, suggest that pragmatic difficulties may be more salient or externally visible among men, which could partially account for higher diagnostic clarity in this group. The relatively low reliance on social camouflaging among autistic men may contribute to a more accurate self‐assessment and visibility of core communicative deficits, aiding differential diagnosis.

Such differences may also be linked to specific cognitive mechanisms associated with ASD, such as difficulties in interpreting nonliteral meaning and intentional nuances in language—especially irony and humour—which have been more frequently documented in males with ASD (Kaland et al. 2002; Wang et al. 2006). Compared to women, autistic men typically report less sophisticated compensatory strategies, oriented more toward controlling behaviour than simulating social reciprocity (Hull et al. 2019). This may result in a more transparent clinical presentation, making standardized tools such as the PAQ (Rodríguez Muñoz 2012) more reliable in male populations than in females or gender‐diverse individuals.

Finally, for participants with diverse gender identities, no statistically significant differences were found between the ASD and BPD groups. Although research on camouflaging among transgender, nonbinary and gender‐fluid individuals is still emerging, recent studies suggest that masking strategies in these populations may be less related to core features of any specific diagnosis and more closely tied to contextual experiences of invisibility, stigma or social pressure to conform to binary communicative norms (Strang et al. 2020). From this perspective, the absence of differences may indicate that camouflaging in this group is more strongly driven by shared social and identity‐related factors than by diagnostic traits specific to ASD or BPD. This may help explain the score convergence observed in both instruments and calls for diagnostic tools that consider gender‐diverse experiences.

In the PAQ results, only one statistically significant difference was found between ASD and BPD in gender‐diverse individuals—specifically in the item assessing superstructural coherence. No significant differences were observed in the remaining dimensions. One possible explanation is that camouflaging in neurodivergent individuals with diverse gender identities may adopt hybrid forms, combining elements of emotional regulation (associated with BPD) and social simulation (typical of ASD), thereby complicating clinical differentiation in global self‐report measures. Further research is needed to develop adapted tools for evaluating communication in gender‐diverse populations, using multimodal methods and inclusive normative data.

## 5 Clinical Implications

The present findings have several implications for clinical practice. Clinicians should interpret apparent communicative competence with caution and avoid relying solely on surface‐level performance when making diagnostic judgements. A careful examination of developmental history, consistency of functioning and emotional context is essential to differentiate between adaptive camouflaging and underlying pragmatic difficulties.

Assessment procedures should therefore adopt flexible and individualized approaches that account for gender‐related variations in masking and expression, as well as the potential overlap in camouflaging strategies across diagnostic categories such as ASD and BPD. Integrating subjective self‐reports with developmental interviews, behavioural observations and contextual information may lead to more accurate and sensitive diagnostic practices, reducing the likelihood of misclassification—particularly among women and gender‐diverse individuals whose adaptive behaviours may obscure core traits.

## 6 Limitations and Future Directions

This study presents several limitations that warrant consideration. Although the overall sample size was adequate for initial analyses, the subsample of gender‐diverse individuals was relatively small, limiting the generalizability of findings. Additionally, the use of self‐report questionnaires, while valuable for capturing participants' subjective perspectives, may be subject to biases such as self‐perception distortion or social desirability—especially in populations that habitually rely on camouflaging strategies. These limitations are particularly relevant given the recruitment strategy, which relied on online dissemination through social media platforms, potentially introducing selection bias and limiting the representativeness of the sample.

The cross‐sectional design precludes causal inference, and potential confounding variables—such as educational background, therapeutic history, or sociocultural context—were not controlled. For instance, greater education or therapeutic exposure may enhance metacommunicative awareness, thereby influencing self‐assessment. Similarly, certain sociocultural environments may shape communicative styles, influencing the extent to which camouflaging is employed.

Moreover, comorbid mental health conditions (e.g., anxiety, depression, ADHD and trauma‐related disorders) were not systematically assessed or controlled. Given their high prevalence in both ASD and BPD, such comorbidities may influence pragmatic functioning and camouflaging behaviours. Future studies should therefore include standardized clinical screening for co‐occurring disorders to improve diagnostic specificity and interpretative precision.

It is also important to note that the instruments used (CAT‐Q and CCP), while validated for autistic populations, were not specifically developed for individuals with BPD or adapted for gender diversity. This may limit their discriminative power across clinical profiles. In the case of BPD, for example, affective factors such as fear of abandonment may shape communicative styles in ways that generate verbal ambiguity, impacting both interaction and self‐evaluation. Previous studies (Muriel et al. 2023) have already identified specific difficulties in using the CCP with BPD populations, highlighting the need for caution when interpreting results in nonautistic clinical profiles.

A further limitation concerns the exclusive use of self‐report measures, which do not capture the behavioural or interactional aspects of pragmatic communication. Future research should combine these subjective measures with objective or performance‐based assessments such as discourse analysis, conversational tasks or experimental paradigms that evaluate pragmatical inference (e.g., irony or indirect request). Incorporating multiple methods would allow a fuller and more ecologically valid understanding of pragmatic functioning across neurodiverse and clinical populations.

Taken together, these findings underscore the need for further research into the differential diagnosis of ASD and BPD through longitudinal designs, mixed‐method approaches and larger, more diverse samples. Only through such efforts will it be possible to develop more accurate, context‐sensitive and inclusive clinical assessments.

## Ethics Statement

This study was conducted in accordance with the Declaration of Helsinki. Ethical approval was obtained from (CEIS#2025‐103620). Written informed consent was obtained from all participants prior to their inclusion in the study.

## Conflicts of Interest

The authors declare no conflicts of interest.

## Data Availability

The data that support the findings of this study are not publicly available and are not shared due to ethical and confidentiality considerations.
